# MRI-Based Effective Ensemble Frameworks for Predicting Human Brain Tumor

**DOI:** 10.3390/jimaging9080163

**Published:** 2023-08-16

**Authors:** Farhana Khan, Shahnawaz Ayoub, Yonis Gulzar, Muneer Majid, Faheem Ahmad Reegu, Mohammad Shuaib Mir, Arjumand Bano Soomro, Osman Elwasila

**Affiliations:** 1 Glocal School of Science and Technology, Glocal University, Delhi-Yamunotri Marg (State Highway 57), Mirzapur Pole 247121, India; 2 Department of Management Information Systems, College of Business Administration, King Faisal University, Al-Ahsa 31982, Saudi Arabia; 3 College of Computer Science and Information Technology, Jazan University, Jazan 45142, Saudi Arabia; 4 Department of Software Engineering, Faculty of Engineering and Technology, University of Sindh, Jamshoro 76080, Pakistan

**Keywords:** ensemble approach, brain tumor, convolution neural network, magnetic resonance imaging

## Abstract

The diagnosis of brain tumors at an early stage is an exigent task for radiologists. Untreated patients rarely survive more than six months. It is a potential cause of mortality that can occur very quickly. Because of this, the early and effective diagnosis of brain tumors requires the use of an automated method. This study aims at the early detection of brain tumors using brain magnetic resonance imaging (MRI) data and efficient learning paradigms. In visual feature extraction, convolutional neural networks (CNN) have achieved significant breakthroughs. The study involves features extraction by deep convolutional layers for the efficient classification of brain tumor victims from the normal group. The deep convolutional neural network was implemented to extract features that represent the image more comprehensively for model training. Using deep convolutional features helps to increase the precision of tumor and non-tumor patient classifications. In this paper, we experimented with five machine learnings (ML) to heighten the understanding and enhance the scope and significance of brain tumor classification. Further, we proposed an ensemble of three high-performing individual ML models, namely Extreme Gradient Boosting, Ada-Boost, and Random Forest (XG-Ada-RF), to derive binary class classification output for detecting brain tumors in images. The proposed voting classifier, along with convoluted features, produced results that showed the highest accuracy of 95.9% for tumor and 94.9% for normal. Compared to individual methods, the proposed ensemble approach demonstrated improved accuracy and outperformed the individual methods.

## 1. Introduction

Whenever cells in the brain begin to multiply uncontrolled, a mass-like formation forms, known as a brain tumor [1]. Patients with brain tumors have varying survival rates, depending on the tumor’s size and stage of development. According to their point of origin, brain tumors can be identified as either primary or secondary. Unlike secondary tumors, which form outside, primary brain tumors begin inside the brain. Brain tumors are a type of cancer that can lead to severe neurological and physical symptoms and ultimately, death if not detected and treated early. The detection of brain tumors is a challenging task that requires the expertise of highly trained medical professionals. However, the current methods used for brain tumor detection are time-consuming, costly, and can be inaccurate in certain cases [2]. There are several medical imaging modalities that are commonly used to locate and identify a brain tumor. These include magnetic resonance imaging (MRI), magnetic resonance spectroscopy (MRS), and computed tomography (CT). There is an assortment of aspects, such as the suspected tumor kind and location, that should be taken into account when determining which imaging modality to use, based on the patient’s medical history and the availability of imaging equipment. In some cases, multiple imaging modalities may be used to obtain the accurate diagnosis. Therefore, there is a need for a more efficient and accurate method for brain tumor detection.

Artificial intelligence (AI) and machine learning (ML) [3,4,5] have shown tremendous potential in various medical applications [6], including the detection of brain tumors. AI and ML algorithms can analyze large amounts of medical data, such as CT and MRI scans, and accurately identify the presence of brain tumors [7]. They can also provide additional information, such as the type of tumor and its location, which can aid in the treatment planning process. This research has resulted in experimenting with various algorithms and models that can accurately detect brain tumors. However, there are still numerous challenges that need to be addressed, such as improving the accuracy of these algorithms and ensuring their reliability in clinical settings.

Deep neural networks (DNNs) have deep learning, which has revolutionized different areas, such as agriculture [8,9,10,11,12], education [13], finance [14], healthcare [15] and more. Deep learning networks are effective in brain tumor detection and diagnosis because they can automatically learn and extract features from large amounts of brain medical imaging data [16]. DNNs are a particular type of ML algorithm that are intended to replicate the brain’s framework and the function of the human brain. To learn and extract more sophisticated characteristics from the input data, they are built of progressively more complicated layers of interconnected nodes [17,18].

In the context of brain tumor detection and diagnosis, DNNs can analyze large volumes of medical imaging data and automatically identify patterns and features that are indicative of brain tumors. These features may include the shape, size, and location of the tumor, as well as its relationship to surrounding brain tissue. One of the key advantages of DNNs is their ability to learn from vast amounts of data. By training on large datasets that contain both tumor and non-tumor images, DNNs can learn to identify subtle differences between the two and accurately distinguish between them. This ability to learn from large amounts of data also makes DNNs more robust to variations in imaging conditions, such as differences in scanner hardware or patient positioning. Another advantage of DNNs is their ability to incorporate multiple imaging modalities into their analysis. For example, a DNN can integrate information from medical images to improve its accuracy in detecting and diagnosing brain tumors. Overall, DNNs are effective in brain tumor detection and diagnosis because they can automatically learn and extract features from large volumes of brain medical imaging data, and they can integrate information from multiple imaging modalities to improve their accuracy.

CNNs are designed to process images and can automatically learn and extract features from brain imaging data to enhance their accuracy in detecting and diagnosing brain tumors [19]. The features in brain imaging data are analyzed in CNNs through a series of convolutional layers [20,21,22]. Each convolutional layer has a collection of filters that convolutionally float across the input image. These filters detect edges, corners, and textures. Each convolutional layer produces feature maps that show where these features are in the input image. To learn higher-level characteristics more pertinent to the goal of brain tumor identification and diagnosis, the feature maps are then sent through one or more fully connected layers after the convolutional layers. These fully linked layers employ convolutional layer characteristics to predict brain tumor presence or absence. During the training phase, the CNN learns to identify patterns in the brain imaging data that are indicative of brain tumors. This is performed by adjusting the weights of the filters in the convolutional layers and the fully connected layers through a process called back-propagation. By adjusting the weights of the network in this way, the CNN learns to identify patterns in the brain imaging data that are associated with brain tumors. These patterns may include the shape, size, and location of the tumor, as well as its relationship to surrounding brain tissue. Overall, CNNs use convolutional layers to analyze the features in brain imaging data and learn patterns that are indicative of brain tumors. By adjusting the weights of the network during training, CNNs can learn to accurately detect and diagnose brain tumors in medical images.

There have been many attempts carried out by researchers to improve the accuracy of identifying brain tumors. In assessing the clinical importance of each MR sequence in brain tumor prediction and detection, the prior studies are highly helpful and are a great resource. Imaging modalities, such as MRI and CT, were utilized by the researchers in order to discern between tumors and other abnormalities. MR spectroscopy has been employed for the categorization of brain tumors in a wide variety of studies. In particular, spectroscopic and magnetic resonance imaging were used in a study; Wang et al. [23] tried to differentiate benign from malignant brain neoplasms by applying a decision tree algorithm. In contrast, spectroscopic and perfusion magnetic resonance imaging were used in the study by Weber et al. [24] to evaluate the inherent heterogeneity of brain neoplasms by defining four regions of interest in the tumoral and peritumoral regions. The SVM classifier was used by Qin et al. [25] to detect brain tumors. In the first step, characteristics of MR images of brain tumors were extracted using the HOG algorithm and then compared to those extracted using wavelet transform. As a second classification method, they employed SVM with the a-norm loss function. Since it was sparse, it could be detected much more quickly. Several learning algorithms, including the Gaussian mixture model (GMM), the Random Forests classifier (RFC), and the K-means clustering and its derivatives, were presented for the diagnosis of brain tumors by Gopal et al. [26]. The support vector machine was tried and tested by Mathew et al. [27] and Amin et al. [28]. The above approach is a well-solved problem for identifying brain tumors, although it can be made more computationally efficient. Parallel to this, deep learning models have proven to be more effective. Due to the nature of the deep network model architecture, deep learning techniques such as convolutional neural networks (CNNs) need a substantial amount of training data consisting of images of brain tumors [29].

Devnath et al. [30] proposed an ensemble approach with deep features from CheXNet-121, which enhanced pneumoconiosis detection in chest X-rays (CXRs), achieving 92.68% accuracy, 85.66% MCC, and 0.9302 PR AUC, surpassing single model and other techniques; Grad-CAM visualization confirmed over 90% detection accuracy. Devnath et al. [31] introduced ensemble techniques for automating pneumoconiosis detection in coal miners through chest X-rays, achieving a robust accuracy of 91.50% by combining deep learning models, surpassing existing methods. Saeedi et al. [32] trained many ML models to classify brain tumors and achieved accuracies from 95.63% to 96.47%. Sobhaninia et al. [33] proposed a model which simultaneously segmented and classified the brain tumor. Their model achieved an accuracy of around 96.27 for classifying brain tumors, whereas it achieved 97.98% accuracy for segmentation. Zulfiqar et al. [34] proposed a model based on EfficientNet for classifying brain tumors based on MR images. They incorporated transfer learning to improve the accuracy of the proposed model. They further added some layers to enhance the model in terms of accuracy and achieved 98.86% accuracy. In another study [35], authors tried to predict the age of the patients with malignant brain tumors. They first performed the segmentation and then performed feature extraction. They used MRI brain images and improved the accuracy by 33%, compared to existing research works.

This study introduces a novel system that offers several advantages in the context of brain cancer forecasting. The main contributions and characteristics of the proposed system are as follows:

- Ensemble model with convolutional features: The research proposes an ensemble model that combines convolutional features extracted from a specialized convolutional neural network (CNN). The ensemble model incorporates a voting mechanism, employing logistic regression and a stochastic gradient descent classifier to generate a final prediction.
- Comparison of convolutional features: The effectiveness of models utilizing convolutional features is compared to the impact of using the original characteristics of the data. This analysis provides insights into the benefits and performance improvements offered by the convolutional features in the context of brain cancer forecasting.
- Evaluation of multiple ML models: The study evaluates the performances of various machine learning (ML) models, including Random Forest (RF), K-Nearest Neighbor (k-NN), decision tree (DT), and Extreme Gradient Boost (XG-Boost). The results of these models are compared to assess their effectiveness in the classification task.
- Ensemble model experimentation: Among the evaluated ML models, the three best-performing models are selected for further experimentation as an ensemble model. This ensemble approach aims to leverage the strengths of multiple models to improve the classification accuracy of the system.
- Performance comparison with other ML methods: The proposed framework’s performance is assessed in terms of accuracy, precision, memory usage, and the F1-score. The evaluation includes a comparison with other ML methods to determine the superiority of the proposed framework in the context of brain cancer forecasting.

By employing an ensemble model with convolutional features and conducting a comprehensive evaluation of various ML models, this work provides insights into the effectiveness and performance of different approaches for brain cancer forecasting. The proposed framework’s performance is thoroughly assessed and compared to other ML methods, providing a comprehensive analysis of its capabilities.

## 2. Materials and Methods

**Figure 1:**
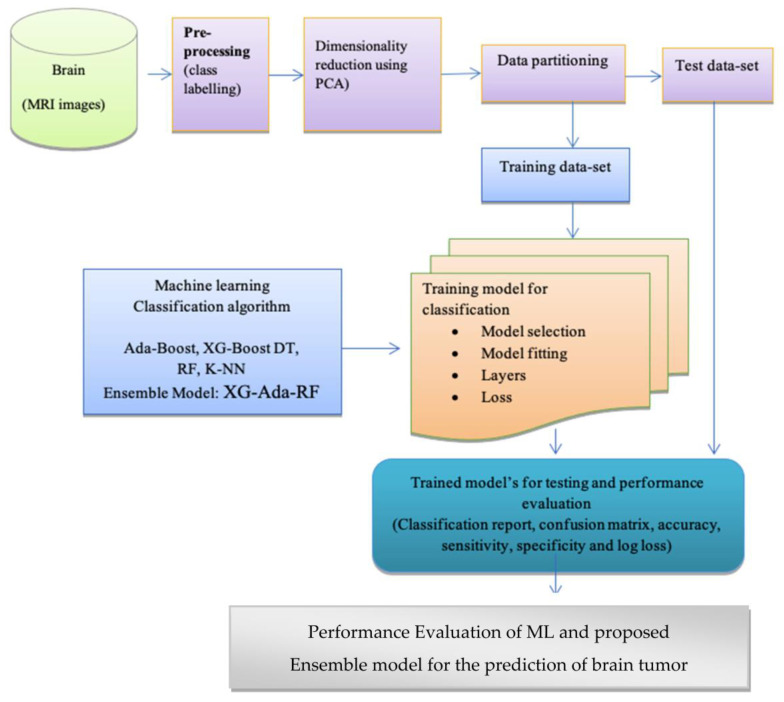
Workflow diagram of the proposed methodology.

In this section, the proposed framework for brain tumor prediction is discussed using the MRI dataset, as shown in Figure 1. The ML classifiers utilized in this work are also briefly described in this section.

The convolutional operations of CNN are initially utilized in order to extract the features. In addition, a dimensionality reduction method is then used to pick a limited collection of effective features for classification in order to increase the generalization ability and performance of the classifier. This is performed in order to make the classifier more efficient. The dataset and the data will be discussed in this part of the article.

After that, the pre-processing of the data, feature extraction, and dimensionality reduction are detailed, and then the classification algorithms that were employed are presented.

### 2.1. Dataset Description

**Figure 2:**
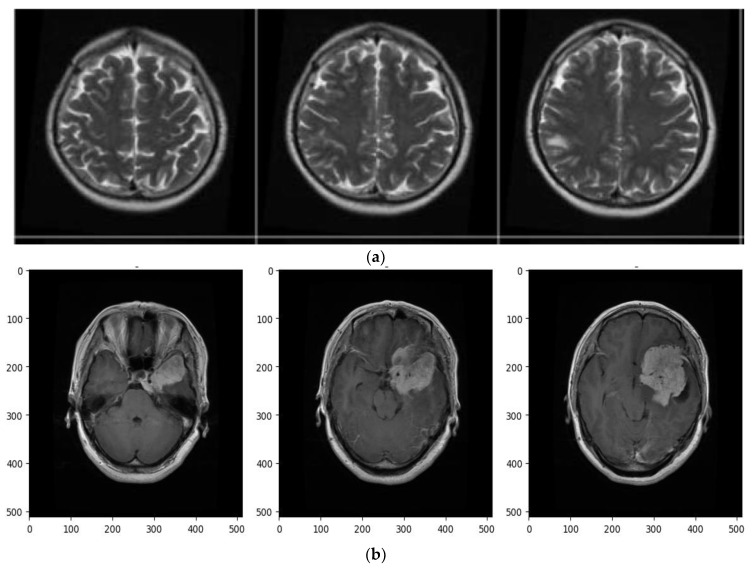
(**a**) Sample MRI 2D images for normal brain. (**b**) Sample MRI 2D images for tumor brain.

This study made use of the “Brain tumor” dataset, which is publicly available on figshare [36]. The dataset contained 3762 instances. The target features contained two classes: tumors and non-tumor, of which 2079 belonged to the non-tumor class (Healthy), and 1683 belonged to the tumor class. Class-wise distributions of the sample are given in Table 1. Machine learning and deep learning models require a good amount of data to train them [37]. From Table 1, it can be seen that the dataset was balanced. A few sample images of brain tumors and normal images are displayed in Figure 2. Input data from the MRI scans were transformed into NumPy arrays and used by the model. A NumPy array is an n-dimensional array that can be expressed by a grid of values with non-negative number tuples.

### 2.2. Convolutional Neural Network for Feature Engineering

Conventional ML algorithms can be effective in detecting brain tumors from imaging data by learning patterns from large datasets. Feature extraction and selection are critical steps in developing a successful machine learning model, and various approaches can be used to identify relevant features for training the model [38]. Algorithms are trained to differentiate between images of a healthy brain and a brain with a tumor, based on various features, such as shape, +size, texture, and intensity [39]. Feature extraction and selection are crucial steps in developing a machine learning model for detecting brain tumors. In this context, feature extraction refers to the process of identifying relevant features from the imaging data, while feature selection involves choosing the most informative subset of features that can help the model perform well [40].

### 2.3. Principal Component Analysis (PCA)

There are various approaches to feature selection in ML algorithms for brain imaging. One common approach, such as PCA, is to reduce the dimensionality of the data and extract relevant features [41]. Another approach is to use domain-specific knowledge to select features that are known to be relevant for tumor detection, such as the size and shape of the tumor or the intensity values of the image. Once the features have been extracted or selected, they are used as inputs to train a machine learning model. During the training process, the model adjusts its parameters to learn the relationship between the input features and the corresponding output (i.e., tumor or no tumor).

**Figure 3:**
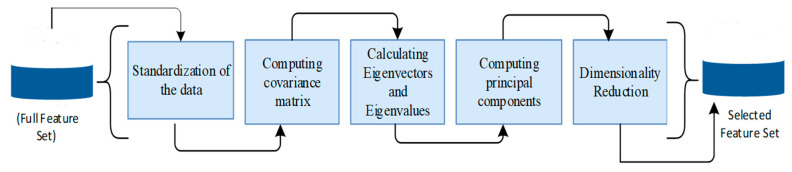
Step-by-step procedure of PCA for dimensionality reduction.

After pre-processing, the number of features obtained was more than 23,000 for a set of 3712 brain MRI images. Therefore, optimization was performed using PCA to overcome the curse of dimensionality, removing data inconsistencies and redundant data and finding correlations among different features to create a new data space with fewer features that retain most relevant features. After using PCA, 12,000 features were selected to reduce the dimension for better performance. The stepwise functionality of PCA is represented in Figure 3. PCA transformed the data into a set of significantly lower dimensions without the loss of relevant information that accounted to final output.

### 2.4. Methods

The following machine learning models were explored as part of this work to perform analyses for improved brain tumor prediction using MRI data.

#### 2.4.1. Adaptive (Ada-Boost) Boosting

Ada-Boost is one of the earliest ensemble-boosting algorithms [42]. The weighting instances of the dataset serve as Ada-Boost’s framework. It begins each observation with the same weights. The method is trained using the observation’s weights, and a feeble classifier is produced. Using the coefficient, we evaluated the performance of the limb. When data were incorrectly categorized, weights were increased, and when data were appropriately categorized, weights were decreased. This procedure was repeated to generate a classifier for weighted data using the weak learning method.

#### 2.4.2. Extreme Gradient (XG-Boost) Boosting

XG-Boost is an extremely effective and adaptable method for distributed gradient boosting. XG-Boost is a framework for gradient boosting that integrates machine learning classifiers [43]. It employs a parallel tree-boosting technique to manage a variety of machine learning issues efficiently and effectively. During training and testing, the XG-Boost classifier is extremely fast. In addition, the regularization parameter effectively reduces variance, thereby improving model performance.

#### 2.4.3. The K-Nearest Neighbor (K-NN)

Altman first introduced the non-parametric technique for data classification in 1991 [44]. Initially, the K-NN method was designed to address both classification and regression issues [45]. Similarity measure, also known as closeness, proximity, or distance, was the guiding principle behind the development of K-NN.

#### 2.4.4. Decision Tree (DT)

In the category of non-parametric supervised learning is the decision tree classifier. The decision node and the root node are the two branches that emerge from the node to serve the inner node [27]. The leaf nodes represent pure subsets that are generated in accordance with the decision rule.

#### 2.4.5. Random Forest (RF)

Friedl et al. [45] created the Random Forest classifier in 1997 by combining multiple decision trees. This technique of ensemble learning employs the bootstrap bagging or aggregation technique to trees [46]. Using arbitrarily selected data (a subset of data), the RF classifier generates a cluster of trees by aggregating the results of several trees, or decision trees. The final classification of the input is determined by combining the results of multiple decision trees and aggregating the majority ballots from various decision trees.

## 3. Proposed Novel Ensemble Approach: XG-Boost_Ada-Boost Random-Forest (XG-Ada-RF)

**Figure 4:**
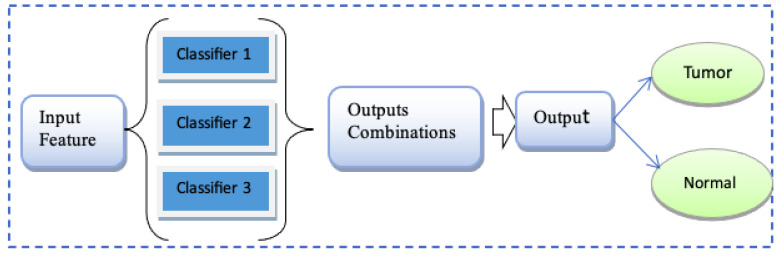
Ensemble voting.

Ensemble is the idea of wrapping up the basic models to make the average prediction, which is more reliable in comparison with a single prediction with single models [47]. It works based on the majority voting. The ensemble voting scheme has made it so that error due to the independent classifier is overcome [48,49]. The ensemble technique offers improved accuracy by using intelligent computational models, as illustrated in the Figure 4. Each model contributes its expertise, and the ensemble aims to leverage the strengths of each individual model while compensating for their weaknesses.

**Figure 5:**
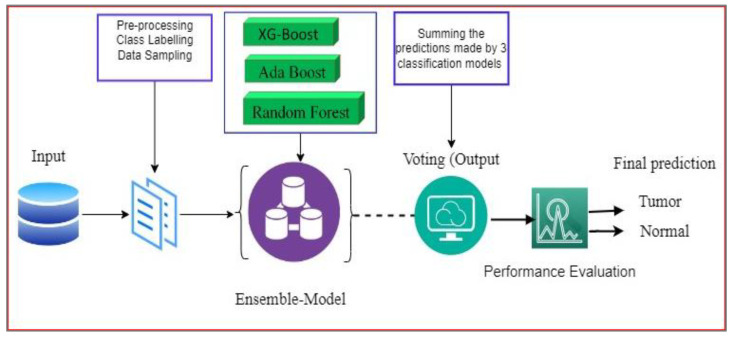
Proposed ensemble model (XG-Ada-RF).

This study applied an ensemble Ada-Boost, XG-Boost, and Random Forest (XG-Ada-RF), based on their individual performances for the binary classification of brain MRIs for predicting brain tumors. The detailed architecture of the proposed ensemble XG-Ada-RF model is shown in Figure 5. The pseudocode for the proposed study is given in Algorithm 1. The ensemble approach increases the performance by combining the advantages of more than one classifier.

The first step involves training three individual machine learning models: XG-Boost, Ada-Boost, and Random Forest. Each model is trained on the same brain tumor classification dataset, using a combination of labeled brain MRI images and corresponding tumor labels. Once the individual models are trained, they are used to make predictions on the test data. Each model generates its set of predictions, indicating whether each test image is classified as a tumor or normal. The ensemble combines the predictions from the three individual models using a weighted voting approach. Each model’s prediction is assigned a weight based on its performance and reliability during the training phase. The models with higher accuracy or lower error rates may receive higher weights, indicating their greater influence on the ensemble’s final decision. The ensemble aggregates the weighted predictions from the individual models. This can be performed using a simple majority vote, where the class with the most votes is considered the final prediction. The aggregated prediction is considered the ensemble’s final decision for each test image. This decision is based on the combined expertise of the individual models, taking into account their diverse decision-making processes and generalization capabilities.
**Algorithm 1:** Procedure Ensemble Model (XG−Ada−RF)**Input:** The training dataset ${(d}_{train})$, the test dataset ${(d}_{test})$, the input shape (inp) **Output:** The output is classified in two categories: normal and brain tumor, and the model will return the results based on accuracy, precision, recall, fbeta_score 1: Data_Preprocessing, Class labelling, Data-resampling 2: $Feature{Extraction}_{CNN}\leftarrow ConvolutionLayers$ 3: model_cnn.add_MaxPooling2D(pool_size, stride) 4: model_cnn.add(Dropout(rate)) 5: model_cnn.add(Flatten()) 6: models_(Ada−Boost,XG−Boost,DT,KNN,RF) 7: Proposed_Ensemble_model_XG−Ada−RF() 8:$\;accuracy,precision,\;recall,\;fbeta\_score,auc\leftarrow model\_Evaluate(d_{test})$ 9: Return accuracy,precision, recall, fbeta_score, auc


### Experimental Environment Settings and Performance Evaluation Metrics

The experimentation setup required a 64 GB RAM Intel i7 11th Generation processor and an 8 GB Nvidia RTX3070 GPU. For parallel computing, 16 cores were utilized using threaded gradient descent. Tensorflow 3.1 and Keras were used as the backbones, providing the modeling libraries. Aside from the hardware requirements, Anaconda, Python, and VS Code were used to boost additional processing power. After the preprocessing phase was completed, the image volumetric blob was passed to the sparse volume convolution to increase the channeling output.

Several metrics were employed to evaluate the performances of the models, including accuracy, precision, recall, and F1-score, which are frequently used indicators.

(1) $$Accuracy=\frac{True\;Positive+True\;Negative}{True\;Positive+True\;Negative+False\;Positive+False\;Negative}$$

(2) $$Recall=\frac{True\;Positive}{True\;Positive+False\;Negative}$$

(3) $$Precision=\frac{TP}{True\;Positive+False\;Positive}$$

(4) $$F1-Score=2\times \frac{Recall\times Precision}{Recall+Precision}$$

## 4. Results and Discussions

The objective of this study is to identify the effectiveness of different algorithms for accurate classification and with minimum log loss. Accuracy represents the degree to which a measurement corresponds to its actual value. Sensitivity mathematically represents the values by calculating the number of correct positive predictions divided by the total number of positive values. Specificity mathematically represents the value by calculating the number of correct negative predictions divided by the total number of negative values. Sensitivity and specificity are statistical measures of the performance of the test results.

**Figure 6:**
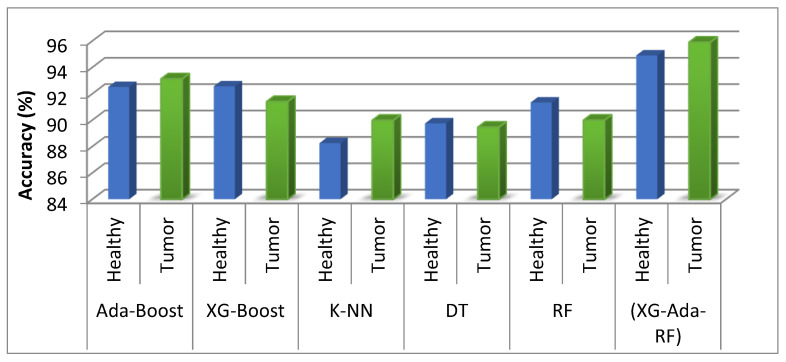
Performance comparison for brain tumor classification binary class for each model implemented in this work.

Table 2 displays the classification results, including accuracy, sensitivity, specificity, and loss, for the various classification algorithms reported in this study. Different classification accuracy performances on testset were observed for various algorithms, based on the observed results. Several machine learning and proposed ensemble models were applied on the brain MRI dataset for the classification, but out of them all, the proposed approach stood out from other models. Upon comparing the performance evaluations of ML and EL techniques, Ada-Boost yielded a great accuracy of 93.12% among all models and also attained great results in terms of precision, recall, and F1-score, with the lowest loss compared to other models. The three best-performing learners were than combined in an ensemble approach, such as Ada-Boost, XG-Boost, and Random Forest, which performed great on the dataset, in contrast to machine learning models. The ensemble proposed model made better predictions. The graphical representation for comparing the performance of each model is shown in Figure 6.

**Figure 7:**
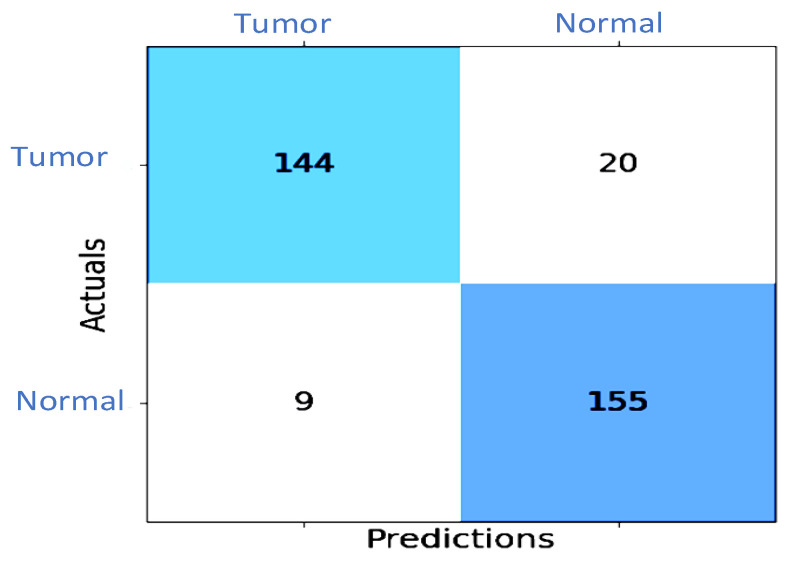
Confusion matrix for the XG-Ada-RF model.

**Figure 8:**
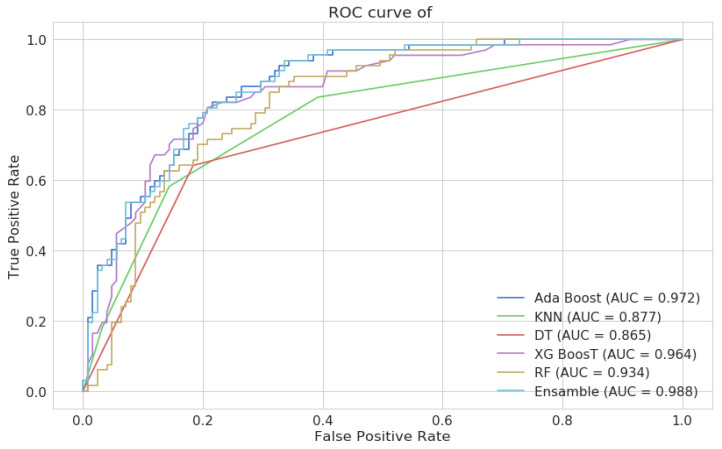
ROC for all the implemented models and the proposed XG-Ada-RF model.

The efficacy of the proposed XG-Ada-RF model was evaluated using a 2 × 2 confusion matrix, where 2 is the total number of target classes. As shown in Figure 7, the matrix compared the actual target values to those predicted by the XG-Ada-RF model. The receiver operating characteristic curve (ROC) plots are given in Figure 8.

To detect brain tumors early, the study employed brain MRI data and effective learning paradigms. CNNs have made tremendous advances in visual feature extraction tasks, making them suitable for our goal. By utilizing deep convolutional features, we aimed to enhance the precision of brain tumor and non-tumor patient classifications. These learned features conveyed critical visual information, allowing the model to detect tiny brain tumor patterns and fluctuations. Authors experimented with five ML models to broaden our understanding of their capabilities and significance in brain tumor classification. The models evaluated Extreme Gradient Boosting, Ada-Boost, and Random Forest, among others. We compared their individual performances to identify the most promising candidates for our proposed ensemble approach. An ensemble of three high-performing individual ML models, Extreme Gradient Boosting, Ada-Boost, and Random Forest (XG-Ada-RF), was proposed. The ensemble approach aimed to leverage the strengths of these individual models while mitigating their weaknesses. The ensemble took advantage of the diverse behaviors and decision-making processes of the constituent models, resulting in more robust and accurate predictions. The proposed ensemble approach achieved a high level of accuracy in binary class classification for detecting brain tumors, and the results validated the significance of the ensemble approach.

Furthermore, we compared our study with the techniques used in previous studies, as shown in Table 3. The table encapsulates a compilation of studies concerning brain tumor classification via MRI scans. Each study employed distinct methodologies to attain accurate results. One study employed a neural network with a back-propagation technique, achieving a high accuracy rate. Another study adopted a Naïve Bayes classifier approach, obtaining a notable accuracy score. Ensemble learning was harnessed in another study, demonstrating competitive accuracy. The support vector machine (SVM) technique was used in a study, resulting in a remarkable accuracy rate. Additionally, a study combined a saliency map and deep learning strategies, achieving a robust accuracy. Lastly, the proposed model introduced a novel ensemble XG-Ada-RF approach, yielding a commendable accuracy rate. These studies collectively underscored the diverse techniques employed in advancing the field of brain tumor classification through MRI data analysis, with potential implications for medical diagnosis and treatment strategies.

## 5. Conclusions

This paper provides a comprehensive review of the research on the detection and classification of brain tumor MRI images using ML algorithms, specifically focusing on the use of an ensemble of the three best-performing models. Through a critical analysis of conventional ML models, this study offers a detailed examination of their strengths and limitations. The proposed ensemble model, XG-Ada-RF, demonstrates superior predictive performance, with an accuracy of 94.9% for the healthy class and 95.9% for the tumor class. This outcome highlights the effectiveness of the ensemble approach in accurately predicting and classifying brain tumors from medical image data. Building upon this research, a comprehensive framework based on an ensemble of three classifiers was developed to achieve accurate predictions and classifications. This framework serves as a valuable tool for enhancing our understanding and expanding the scope and significance of brain tumor classification.

Furthermore, an experimental comparative performance analysis was conducted to assess the robustness of the various algorithms and the ensemble model. Key evaluation metrics, such as accuracy, sensitivity, specificity, and log loss, were considered to provide a comprehensive evaluation. The diverse results obtained by implementing the aforementioned algorithms on the given dataset further supported the objectives of this study.

Looking ahead, future research directions could focus on refining and optimizing the ensemble model, exploring additional ML algorithms, and incorporating advanced techniques, such as deep learning. Additionally, further investigation into the interpretability and explaining ability of the ensemble model could provide valuable insights for clinical decision-making. Overall, the results of this study emphasize the superiority of the ensemble approach in brain tumor classification, laying a foundation for further advancements in this field.

## Figures and Tables

**Table 1:** Detailed sample-wise distribution of the dataset.

Dataset	Normal	Brain Tumor
Brain Images	2079	1683

**Table 2:** Performance evaluation of classifiers for brain tumors.

Model	Class	Accuracy (%)	Precision	Recall	F1-Score	AUC
**Ada-Boost**	Healthy	92.51	0.8897	0.8897	0.8897	0.9732
	Tumor	93.12	0.8723	0.8556	0.8764	0.9723
**XG-Boost**	Healthy	92.56	0.9360	0.9360	0.9360	0.9821
	Tumor	91.40	0.8764	0.9374	0.9293	0.9635
**K-NN**	Healthy	88.25	0.9327	0.8621	0.9325	0.8861
	Tumor	89.98	0.8734	0.9748	0.8357	0.8767
**DT**	Healthy	89.75	0.7246	0.7119	0.7956	0.8349
	Tumor	89.46	0.8345	0.7897	0.8234	0.8645
**RF**	Healthy	91.33	0.9440	0.9406	0.9433	0.9318
	Tumor	89.99	0.9234	0.8945	0.9234	0.9344
**Proposed Model** **(XG-Ada-RF)**	**Healthy**	**94.9%**	**0.9832**	**0.9423**	**0.9764**	**0.9932**
	**Tumor**	**95.9%**	**0.9888**	**0.9534**	**0.9823**	**0.9878**

**Table 3:** Performance comparison with previous studies.

S. No	Study	Material	Technique	Result (%)
1.	Ismael et al. [50]	Brain MRI	Back-propagation neural network	91.9
2.	Zaw et al. [51]	Brain MRI	Naïve Bayes classifier	94
3.	Kang et al. [52]	Brain MRI	ensemble learning	91.58
4.	Deepak et al. [53]	Brain MRI	SVM	95.82
5.	Khan et al. [54]	Brain MRI	saliency map and deep learning feature optimization	94.89
6.	Proposed Model	Brain MRI	Ensemble XG-Ada-RF model	95.9

## Data Availability

A public dataset was used in this research work [36].
